# Letter to the editor: Increasing proportion of new HIV diagnoses in Ireland previously diagnosed elsewhere – potential impact on estimating incidence

**DOI:** 10.2807/1560-7917.ES.2017.22.9.30472

**Published:** 2017-03-02

**Authors:** Kate O'Donnell, Derval Igoe

**Affiliations:** 1 Health Protection Surveillance Centre, Dublin, Ireland

**Keywords:** Ireland, blood-borne infections, HIV infection, surveillance, epidemiology

**To the editor:** In a recent issue of *Eurosurveillance*, Pharris et al. presented a very interesting study which estimated the HIV incidence and number of undiagnosed people living with HIV in the European Union/European Economic Area (EU/EEA) [1]. The ‘Incidence Method’ used was a CD4 cell count-based back-calculation method [2].

The use of CD4 counts raises important issues for HIV surveillance and interpretation of findings. The Irish surveillance system, for example, includes all persons newly diagnosed with HIV in Ireland, even if previously diagnosed elsewhere. An increasing proportion of newly-diagnosed HIV cases have previously been diagnosed HIV-positive in another country before arrival in Ireland, and have been receiving antiretroviral therapy (ART). In 2015, 27% (129/485) of newly-diagnosed HIV cases had a previous HIV diagnosis in another country, up from a range of 14%–18% for the years 2011 to 2014 [3]. By risk group, heterosexuals were the group with the highest proportion previously diagnosed HIV-positive (35%, 45/130), followed by men who have sex with men (MSM) (29%, 72/247) and people who inject drugs (PWID) (10%, 5/49). The majority (79%, 102/129) of those with a previous HIV diagnosis, transferred their HIV care to Ireland and 63% (81/129) had been receiving ART before arrival in Ireland.

We found that when looking at stage of infection, as per CD4 cell counts at diagnosis, the contribution of people who were previously diagnosed HIV-positive and on treatment can make a considerable difference to the findings. Of those newly diagnosed with HIV in Ireland in 2015, 45% (n=161) were late presenters (CD4 cell count at diagnosis less than 350 cells/µl or an AIDS defining illness at diagnosis), where information on CD4 count or AIDS defining illness at diagnosis was available (74%, 357/485). However, confining the analysis to those who were not reported to have a previous diagnosis abroad, 52% (126/243) presented late. Understandably, the proportion of people presenting late among those who had a previous HIV diagnosis was much lower (31%, 35/114). Consequently, the use of CD4 count data to estimate HIV incidence should be carefully considered in countries where surveillance data includes a significant proportion of people who have been previously diagnosed with HIV in another country, who have previously received ART, and who have transferred their care to Ireland.

Another issue for consideration is the best way to present HIV surveillance data to reflect the increasing proportion of people with prior HIV infection. This will be important in order to accurately measure the impact of HIV prevention strategies within countries and at European level. Due to increased access to testing and treatment worldwide and the mobility of populations in general, this number is likely to remain high. In our opinion this should be an important consideration for surveillance of HIV at both national and European levels.
